# Mid- to long-term rates of symptomatic adjacent-level disease requiring surgery after cervical total disc replacement compared with anterior cervical discectomy and fusion: a meta-analysis of prospective randomized clinical trials

**DOI:** 10.1186/s13018-020-01957-3

**Published:** 2020-10-12

**Authors:** Yifei Deng, Guangzhou Li, Hao Liu, Ying Hong, Yang Meng

**Affiliations:** 1 Department of Orthopedic Surgery, West China Hospital, Sichuan University, No. 37 Guo Xue Xiang, Chengdu, 610041 Sichuan China; 2 Division of Spine Surgery, Department of Orthopedics, Affiliated Hospital of Southwest Medical University, No. 25 Taiping St, Luzhou, 646000 Sichuan Province China; 3 Department of Anesthesia and Operation Center/West China School of Nursing, West China Hospital, Sichuan University, No. 37 Guo Xue Xiang, Chengdu, 610041 Sichuan China

**Keywords:** Adjacent segment disease, Cervical total disc replacement, Anterior cervical discectomy and fusion

## Abstract

**Background:**

Thus far, no meta-analysis focusing on the mid- to long-term incidence of adjacent segment disease requiring surgery after cervical total disc replacement and anterior cervical discectomy and fusion has been published yet. This study aimed to compare mid- to long-term rates of symptomatic adjacent-level disease requiring surgery after cervical disc replacement and anterior cervical fusion.

**Methods:**

A meta-analysis was performed, and only randomized controlled trials with a follow-up period of more than 48 months reporting rates of symptomatic adjacent-level disease requiring surgery after cervical total disc replacement and anterior cervical discectomy and fusion were included.

**Results:**

The analysis revealed that the overall rate of symptomatic adjacent-level disease requiring surgery in the cervical disc replacement group was significantly lower than that of the anterior cervical fusion group at 48–120 months’ follow-up. The subgroup analysis of different follow-up periods also yielded the same results. The rate of symptomatic adjacent-level disease requiring surgery in the cervical disc replacement group using unrestricted prosthesis was significantly lower than that of the anterior cervical fusion group (*p* < 0.001); however, the cervical disc replacement group using semi-restricted prosthesis showed no statistical difference compared with the fusion group.

**Conclusions:**

Our review suggests that cervical disc replacement is preferable to anterior cervical fusion in reducing the incidence of symptomatic adjacent-level disease requiring surgery at mid- to long-term follow-up. A review of the literature also demonstrated that randomized controlled trials investigating the rate of symptomatic adjacent-level disease requiring surgery were insufficient; therefore, studies focusing on this subject with longer-term follow-up are warranted.

## Background

Anterior cervical discectomy and fusion (ACDF) has a long history of successfully treating cervical spondylotic disease and other cervical pathologies, and at present, it is still regarded as a relatively safe and effective treatment option [1–3]. However, ACDF is associated with potential concerns, and one overarching concern is the development of symptomatic adjacent segment disease (ASD) after surgery. Fusion techniques result in altered biomechanics of the cervical spine and potentially accelerate the degenerative process at adjacent segments, which may be one of the primary causes of ASD [4, 5]. ASD was reported to occur at a rate of 2.9% annually in patients who underwent ACDF, with approximately 25.6% of patients predicted to develop symptomatic ASD within 10 years in a landmark study by Hilibrand et al. [1, 5]. Lee et al. performed a large retrospective study and reported similar results [6]. In an effort to address this concern, a variety of non-fusion or motion-preserving techniques, such as cervical total disc replacement (TDR), have been introduced and proposed as an alternative treatment option to ACDF [1, 3, 7].

The early to mid-term clinical outcomes after cervical TDR are better than or non-inferior to that of ACDF; however, there remains controversy with regard to TDR decreasing the rate of ASD development [1, 7–9]. It is reasonable to address such controversy by comparing the rates of ASD development after cervical TDR and ACDF based on mid- to long-term data, since it is too early to observe degenerative changes secondary to altered biomechanics of the non-fused or fused cervical spine using early- to mid-term data [10]. Recently, several 7- to 10-year data from the US Food and Drug Administration investigational device exemption randomized controlled trial (RCT) studies have been published, rendering the analysis of mid- to long-term rates of ASD development after cervical TDR possible [11–13]. However, due to the lack of established standardized criteria, the application of different criteria in studies investigating ASD might be another reason why investigators could not arrive at the same conclusion [14]. Meanwhile, ASD was the most frequent indication for surgery at the adjacent level after TDR and ACDF, but it was not the only indication, since neck pain or revision surgery at the index surgical level might also be considered as such [15–17]. Hence, based on these factors, several meta-analyses reviewing RCTs failed to resolve the issue [10, 18–23]. Consequently, the incidence rate of symptomatic adjacent-level disease requiring surgery (SALDRS) might be a more objective parameter than that of ASD. To date, no meta-analysis focusing on the mid- to long-term incidence of SALDRS after TDR compared with ACDF has been published yet. Thus, this meta-analysis was performed with the aim of comparing mid- to long-term SALDRS rates between TDR and ACDF for the first time.

## Methods

This meta-analysis was conducted in accordance with the PRISMA statement [24].

### Search strategy

PubMed and Embase (updated until July 1, 2019) were searched exhaustively using a combination of the following terms: (“arthroplasty” [title/abstract] OR “replacement” [title/abstract] OR “arthrodesis” [title/abstract] OR “prosthesis” [title/abstract]) AND “cervical” [title/abstract] AND “fusion” [title/abstract]. No language restrictions were applied. We also reviewed the reference lists of relevant studies to search for potentially relevant studies. Repetition of studies was identified according to the study information (such as hospitals and study period). Only the largest one among the studies with patient overlap could be retained.

### Inclusion and exclusion criteria

The inclusion criteria were as follows: (1) RCTs, (2) medically confirmed degenerative cervical spine disease requiring surgical intervention, (3) studies comparing the postoperative incidence of SALDRS between ACDF and TDR, and (4) studies with a follow-up period of > 48 months.

The exclusion criteria were as follows: (1) studies that did not report the incidence of SALDRS in both the ACDF and TDR groups; (2) unrelated research studies; (3) literature reviews or meta-analyses, biomechanical studies, case reports, letters, and conference abstracts; (4) prospective non-randomized studies and retrospective studies; and (5) studies reporting the incidence of SALDRS with the same follow-up period as that of studies in which patient overlap occurred.

### Data extraction

The following required data were extracted independently by two authors: author, year of publication, country, study design, number of patients, follow-up period, and SALDRS. Any discrepancies in the data extracted by the two authors were resolved by a discussion with the third author.

### Risk of bias assessment

To evaluate the quality of the included RCTs, two authors independently evaluated the risk of bias of eligible studies according to the Cochrane Back Review Group Guideline [25]. Any disagreement between the two reviewers would be discussed with the third author to achieve consensus. The items included in the risk of bias assessment were as follows: random sequence generation, allocation concealment, blinding of participants and personnel, blinding of outcome assessment, incomplete outcome data, selective reporting, similarity of baseline indicators, and other biases. Each item was classified as having a low risk of bias, an unclear risk of bias, or a high risk of bias. If at least 50% of the items indicated a “low risk of bias” in one article, then this article would be considered to have a “low risk of bias.” Otherwise, this article would be considered to have a “high risk of bias.”

### Statistical analysis

This meta-analysis was conducted using Review Manager software 5.3 (RevMan 5.3, Cochrane Collaboration). The risk ratio (RR) and 95% confidence interval (CI) were assessed for dichotomous data (SALDRS). *p* values < 0.05 were considered statistically significant. Begg’s and Egger’s tests were performed using STATA 13.0 (StataCorp., TX, USA) to evaluate publication bias when more than 10 studies were included in a meta-analysis. Moreover, trim-and-fill analysis was also conducted to investigate possible publication bias. The *I*^2^ statistic (ranging from 0 to 100%) was used to evaluate the heterogeneity among the included studies. A *p* value < 0.10 was considered statistically significant. An *I*^2^ statistic > 50% was considered to indicate obvious heterogeneity. A random-effect analysis was performed when the *p* value of the chi-square test was < 0.10 or when the *I*^2^ statistic was > 50%. Otherwise, a fixed-effect analysis was performed. Sensitivity analysis was also conducted to examine the included studies individually. It could be beneficial to explain the reason for the high heterogeneity and analyze the effect of one study on the overall result.

## Results

### Identification of the included studies

**Fig. 1:**
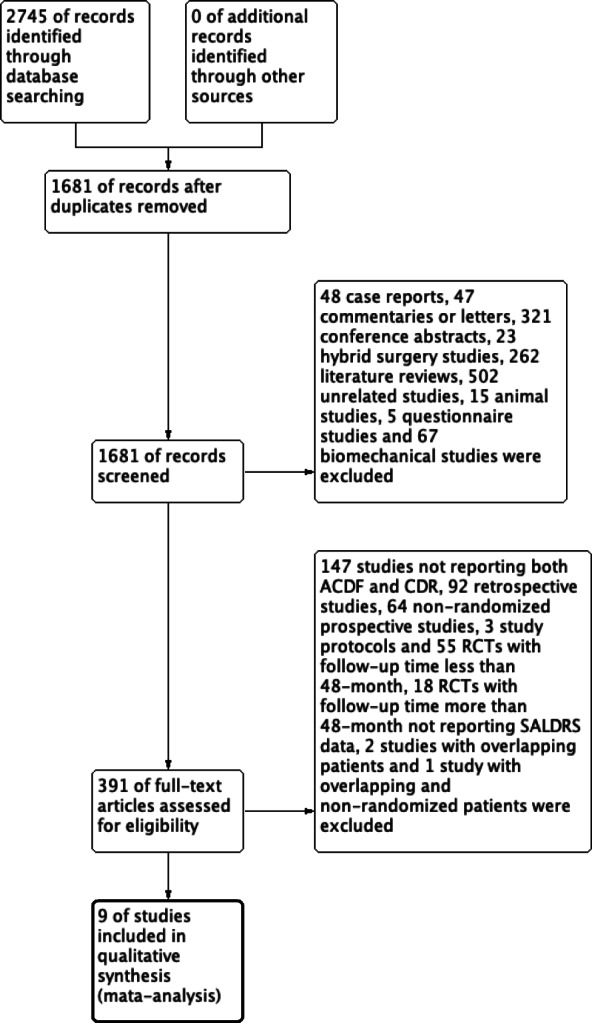
Flow diagram of the study selection

A total of 2745 studies were identified by searching PubMed (*n* = 1118) and Embase (*n* = 1627). Due to duplication, 1064 studies were removed, and 1681 studies were retained. In total, 48 case reports, 47 commentaries or letters, 321 conference abstracts, 23 hybrid surgery studies, 262 literature reviews, 502 unrelated studies, 15 animal studies, 5 questionnaire studies, and 67 biomechanical studies were excluded after assessing the title and abstract. Overall, 147 studies not reporting both ACDF and TDR, 92 retrospective studies, 64 non-randomized prospective studies, 3 study protocols, 55 RCTs with a follow-up period of less than 48 months, and 18 RCTs with a follow-up period of more than 48 months not reporting SALDRS data were excluded after the assessment of full-text articles. There were 12 articles reporting SALDRS data of both the ACDF and TDR groups at 4 years’ follow-up [26–28], 5 years’ follow-up [29–33], 7 years’ follow-up [34–36], 9 years’ follow-up, and 10 years’ follow-up [36, 37]. Only two studies reported data at 4 years’ follow-up, and the follow-up period was close to each other, i.e., between 4 and 5 years. Therefore, the data of studies with 4 and 5 years’ follow-up were combined. Two articles were excluded due to patient overlap [27, 28]. One study [33] was excluded because of patient overlap and non-randomization of some patients in the TDR group. Some studies with patient overlap that reported data at different follow-up periods were included in the subgroup analysis [26, 31]. Ultimately, 9 articles (8 RCTs) were included in the meta-analysis. A flow diagram of the literature search strategy for relevant studies is shown in Fig. 1.

### Characteristics of the included studies

There were 9 articles (8 RCTs) included in the meta-analysis. The basic characteristics of the included studies are presented in Table 1. A total of 2395 patients in 8 RCTs were included in the meta-analysis. There were 1334 patients treated with TDR and 1061 patients treated with ACDF.

**Table 1:** Basic characteristic of the studies included in the meta-analysis

Study	Study design	Sample size		Follow-up time	Prosthesis	Operative level	Follow-up rate		Study center
		TDR	ACDF				TDR	ACDF	
Garrido et al. (2010) [24]	RCT	21	26	48 months	Bryan	1	85.7%	76.9%	Single
Burkus et al. (2010) [28]	RCT	276	265	60 months	Prestige ST	1	52.2%	47.9%	Multiple
Delamarter et al. (2013) [27]	RCT	103	106	60 months	Prodisc C	1	72.7%	63.5%	Multiple
Phillips et al. (2015) [32]	RCT	218	185	84 months	PCM	1	31.2%	22.7%	Multiple
Hou et al. (2016) [30]	RCT	56	51	61 months	Mobi-C	1	91.1%	94.1%	Single
Hisey et al. (2016) [29]	RCT	164	81	60 months	Mobi-C	1	85.5%	78.9%	Multiple
Donk et al. (2017)	RCT	50	47	8.9 ± 1.9 years	Bryan	1	98.0%	97.9%	Single
Radcliff et al. (2017) [33]	RCT	164	81	84 months	Mobi-C	1	80.1%	74.3%	Multiple
	RCT	225	105	84 months	Mobi-C	2	84.4%	75.0%	Multiple
Ghobrial et al. (2018) [34]	RCT	518	486	84 months	Bryan/Prestige ST	1	Not specified	Not specified	Mutiple
	RCT	242	221	120 months	Bryan	1	53.7%	46.6%	Mutiple

*TDR* cervical total disc replacement, *ACDF* anterior cervical discectomy and fusion, *RCT* randomized controlled trial

### Risk of bias assessment

**Fig. 2:**
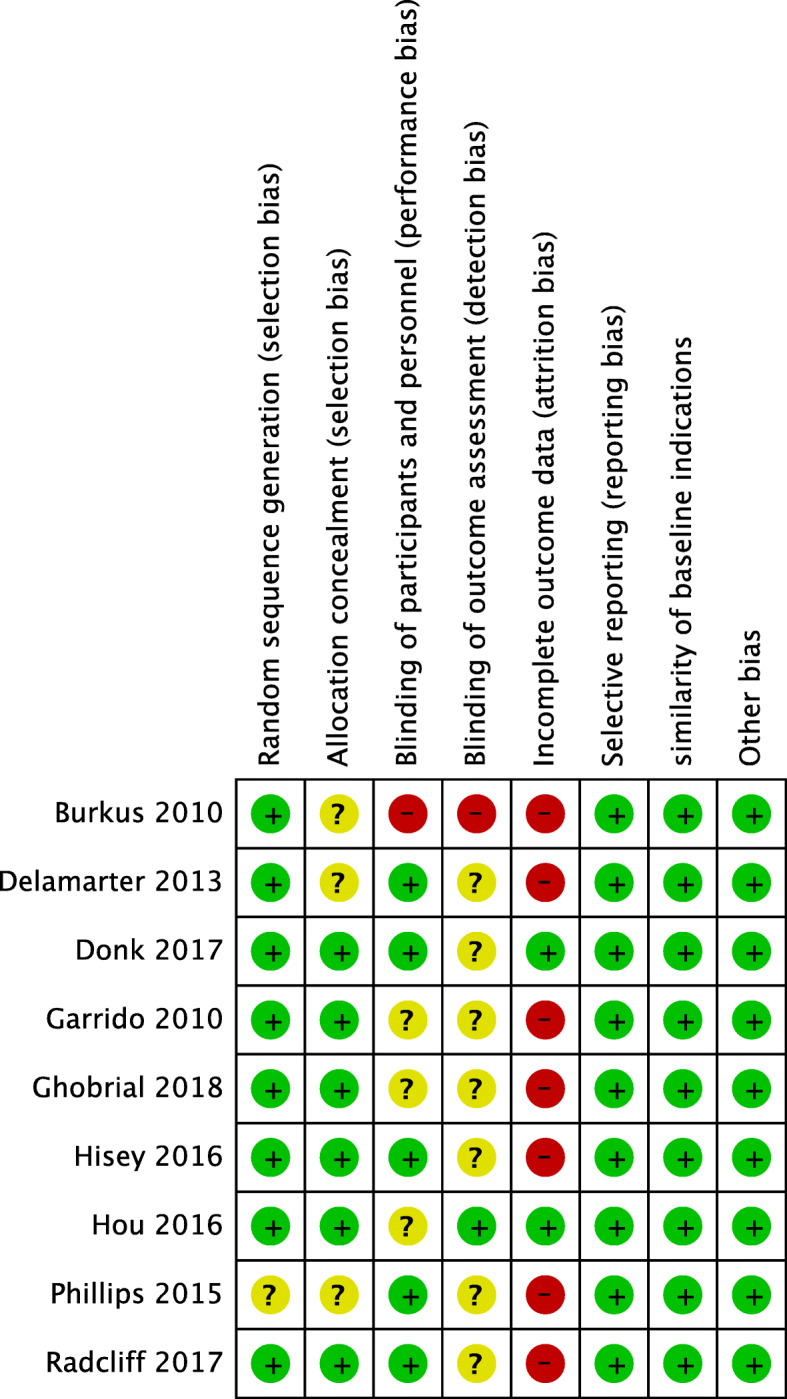
The risk of bias assessment of included studies. Note: +, low risk of bias; −, high risk of bias; ?, unclear risk of bias

According to the Cochrane Back Review Group Guideline [25], all 9 articles were considered to have a low risk of bias (Fig. 2). The majority of studies did not mention allocation concealment and blinding of participants, personnel, and outcome assessment. It was difficult to blind the participants and personnel in studies involving surgical procedures. Most studies showed a high risk of bias in incomplete outcome data. A follow-up rate of over 80% in both groups was observed in only two studies. This study was not eligible for publication bias assessment because only 8 RCTs were included in the meta-analysis.

### Meta-analysis of SALDRS

**Fig. 3:**
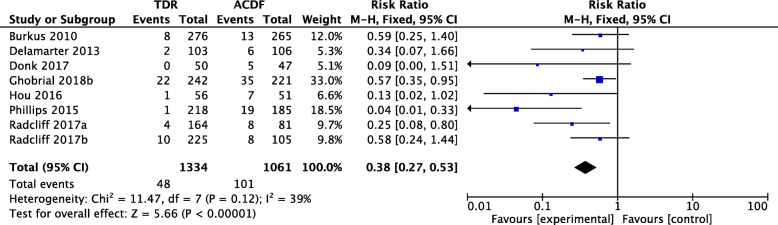
Forest plot comparing the rate of SALDRS between TDR and ACDF. TDR, cervical total disc replacement; ACDF, anterior cervical discectomy and fusion; CI, confidence interval; df, degrees of freedom; M-H, Mantel-Haenszel

Eight studies consisting of 1334 patients in the TDR group and 1061 patients in the ACDF group were included in the meta-analysis. The overall SALDRS rate of the TDR group was significantly lower than that of the ACDF group (RR 0.38, 95% CI [0.27, 0.53], *p* = 0.12, *I*^2^ = 39%, Fig. 3). The heterogeneity was not significant, and the result was reliable. This heterogeneity was attributed to the study by Phillips et al. [34], according to the sensitivity analysis. The SALDRS rate of the TDR group was 0.4% (1/218), while that of the ACDF group was 10.2% (19/185). When the study by Phillips et al. [34] was excluded, the result remained the same (RR 0.46, 95% CI [0.32, 0.65], *p* = 0.50, *I*^2^ = 0%); however, the heterogeneity was lower. This resulted from their unique patient inclusion criteria, in which approximately 12% (29 TDR and 20 ACDF) of the 218 TDR and 185 ACDF patients had prior adjacent or nonadjacent single-level fusions, and the SALDRS rates in the TDR group and ACDF group were theoretically different from those of other RCTs.

**Fig. 4:**
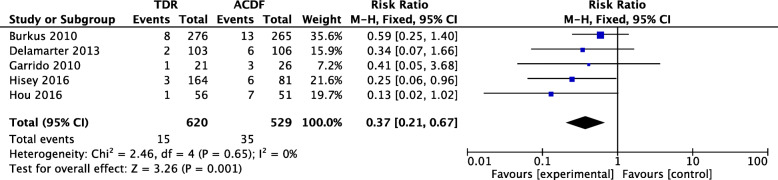
Forest plot comparing the rate of SALDRS between TDR and ACDF at 4–5 years follow-up. TDR, cervical total disc replacement; ACDF, anterior cervical discectomy and fusion; CI, confidence interval; df, degrees of freedom; M-H, Mantel-Haenszel

Five studies with 620 patients in the TDR group and 529 patients in the ACDF group were included in the meta-analysis of the SALDRS rate at 4–5 years’ follow-up. The SALDRS rate of the TDR group was significantly lower than that of the ACDF group (RR 0.37, 95% CI [0.21, 0.67], *p* = 0.65, *I*^2^ = 0%, Fig. 4). The results were considered reliable.

**Fig. 5:**
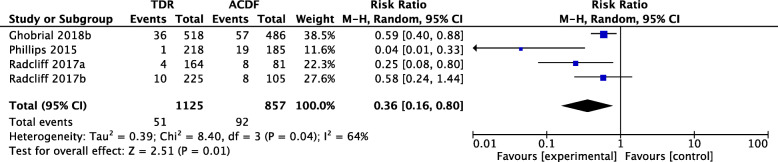
Forest plot comparing the rate of SALDRS between TDR and ACDF at 7 years follow-up. TDR, cervical total disc replacement; ACDF, anterior cervical discectomy and fusion; CI, confidence interval; df, degrees of freedom; M-H, Mantel-Haenszel

Data on the SALDRS rate at 7 years’ follow-up were collected from 4 studies. There were 1125 patients and 857 patients in the TDR and ACDF groups, respectively. The SALDRS rate of the TDR group was significantly lower than that of the ACDF group (RR 0.36, 95% CI [0.16, 0.80], *p* = 0.04, *I*^2^ = 64%, Fig. 5). The heterogeneity was significant, which was attributed to the study by Phillips et al. [34], according to the sensitivity analysis.

**Fig. 6:**
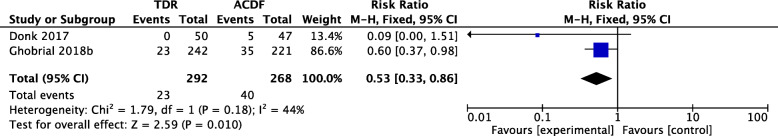
Forest plot comparing the rate of SALDRS between TDR and ACDF at 9–10 years follow-up. TDR, cervical total disc replacement; ACDF, anterior cervical discectomy and fusion; CI, confidence interval; df, degrees of freedom; M-H, Mantel-Haenszel

Two studies consisting of 292 patients and 268 patients in the TDR group and ACDF group, respectively, were included in the meta-analysis of SALDRS rate at 9–10 years’ follow-up. The SALDRS rate of the TDR group was significantly lower than that of the ACDF group (RR 0.53, 95% CI [0.33, 0.86], *p* = 0.18, *I*^2^ = 44%, Fig. 6). *I*^2^ was 44%, which may be caused by the small number of included studies and patients.

**Fig. 7:**
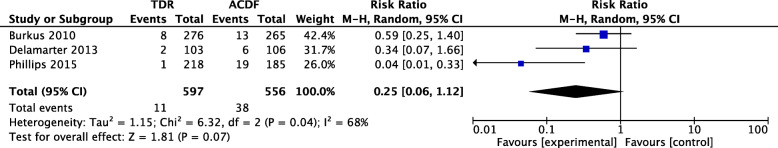
Forest plot comparing the rate of SALDRS between semi-restricted prosthesis TDR and ACDF. TDR, cervical total disc replacement; ACDF, anterior cervical discectomy and fusion; CI, confidence interval; df, degrees of freedom; M-H, Mantel-Haenszel

Data on the SALDRS rate of the groups with semi-restricted prosthesis were collected from 3 studies. There were 597 patients and 556 patients in the TDR and ACDF groups, respectively. The SALDRS rate was lower in the TDR group than in the ACDF group, but no statistical difference was observed (RR 0.25, 95% CI [0.06, 1.12], *p* = 0.04, *I*^2^ = 68%, Fig. 7). Meanwhile, the heterogeneity was significant. After excluding the study by Phillips et al. [34], the heterogeneity changed to 0%. However, the result remained the same.

**Fig. 8:**
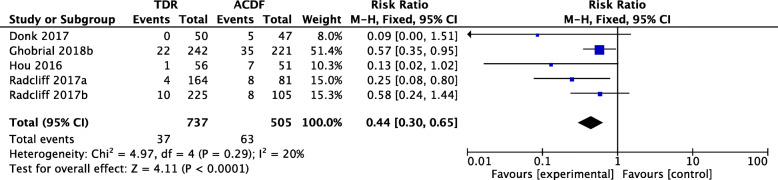
Forest plot comparing the rate of SALDRS between unrestricted prosthesis TDR and ACDF. TDR, cervical total disc replacement; ACDF, anterior cervical discectomy and fusion; CI, confidence interval; df, degrees of freedom; M-H, Mantel-Haenszel

Five studies with 737 patients in the TDR group and 505 patients in the ACDF group were included in the meta-analysis of the SALDRS rate of the groups with unrestricted prosthesis. The SALDRS rate of the TDR group was significantly lower than that of the ACDF group (RR 0.44, 95% CI [0.30, 0.65], *p* = 0.29, *I*^2^ = 20%, Fig. 8). The heterogeneity was low.

### Sensitivity analysis

The sensitivity analysis demonstrated that when the study by Phillips et al. [34] was excluded, the heterogeneity in all meta-analyses changed to 0%. However, the results of all meta-analyses did not change in comparison to the original results.

## Discussion

Despite satisfactory clinical results following TDR, the question of whether TDR can reduce the incidence of ASD is uncertain [1, 7–9]. Some investigators have conducted meta-analyses to resolve this controversy. However, these reviews were unable to reach a consensus [10, 18–23]. The reasons why these studies fail to make an agreement might be as follows: First, there was a lack of sufficient mid- and long-term RCTs, and it was not convincible enough to investigate the incidence of ASD in short- and mid-term RCTs. Second, there was a paucity of consensus on how to define ASD even in RCTs. Third, some reviews focused on reoperation; however, it was the fact that ASD was not the only indication for surgery at the adjacent level, since neck pain or revision surgery at the index surgical level might also be considered as such [15–17]. Thus, it is more reasonable to determine the difference in symptomatic ASD between TDR and ACDF by analyzing mid- and long-term RCTs reporting ASD as the indication for subsequent surgery.

In the present study, we searched the RCTs exhaustively and performed a meta-analysis to compare the mid- to long-term postoperative incidence of SALDRS between ACDF and TDR. We found that the overall SALDRS rate of the TDR group was significantly lower than that of the ACDF group after a minimum follow-up period of 48 months. The heterogeneity was not significant, and the result was reliable. In a meta-analysis, Ren et al. [10] reported that the rate of requiring operation for ASD was not significantly different between patients in the TDR group and the ACDF group; however, the result was derived from only 3 RCTs. Due to the lack of studies reporting on ASD as the indication for operation and the small sample size, Zhang et al. [38] reported that the rate of operations at the adjacent level was not significantly different between the TDR and ACDF groups in their meta-analysis. Wu et al. [15] performed a meta-analysis and compared the overall rates of subsequent surgery and rates of subsequent surgery at the operated level and at the adjacent level between patients who underwent ACDF and TDR. They found that patients in the TDR group had a significantly lower rate of reoperation at the operated level and the adjacent level than those in the ACDF group. However, only 3 of 8 studies in the study by Wu et al. [15] described the reasons for subsequent surgery. Besides, adjacent-level disease was not the only reason for subsequent surgical intervention at the adjacent level. Dong et al. [22] performed a meta-analysis in 2017 and compared adjacent segment reoperation, adjacent segment degeneration, and ASD between patients who underwent ACDF and TDR. They found that the reoperation rate of adjacent levels in the TDR group was significantly lower than that in the ACDF group, and the advantage of TDR in reducing reoperation at the adjacent level increased with increasing follow-up period. However, the difference in ASD between the two groups was not significant. Because of the lack of sufficient RCTs reporting ASD as the indication for operation, neither results of Wu et al. [15] nor Dong et al. [22] were recognized as adequately accurate. The present meta-analysis included a larger number of RCTs reporting ASD as the reason for reoperation; therefore, it is expected to obtain a more accurate and reliable result.

In this meta-analysis, a subgroup analysis with different follow-up periods was performed to investigate the SALDRS rates between the TDR and ACDF groups. The SALDRS rate of the TDR group was significantly lower than that of the ACDF group at 4–5 years, 7 years, and 9–10 years of follow-up. The heterogeneity was not significant, except for the data on the SALDRS rate at 7 years’ follow-up collected from 4 studies. This heterogeneity was derived from the study by Phillips et al. [34], according to the sensitivity analysis. With the exclusion of the study by Phillips et al. [34], the heterogeneity changed to 0%; however, there was no change in the result of the meta-analysis. This is because of their unique patient inclusion criteria, whereby approximately 12% (29 TDR and 20 ACDF) of the 218 TDR and 185 ACDF patients had prior adjacent or nonadjacent single-level fusions, and the SALDRS rates in the TDR group and ACDF group were different from those of other RCTs.

Although the meta-analysis by Wu et al. [15] showed that patients in the TDR group had a significantly lower rate of reoperation at the adjacent level than patients in the ACDF group in RCTs of 48–102 months’ follow-up, there was no subgroup analysis with different follow-up periods in their study. A total of 8 RCTs reporting SALDRS were enrolled in our meta-analysis, and a subgroup analysis with 4–5 years, 7 years, and 9–10 years of follow-up was performed for the first time.

According to previous studies, the different types of prostheses were classified as either unconstrained (Bryan, Mobi-C) or semi-constrained (PCM, Prestige-ST, ProDisc-C) [39, 40]. Artificial discs can be categorized as constrained, semi-constrained, and non-constrained, depending on whether the center of rotation (COR) translates. Constrained implants have a fixed COR, whereas unconstrained implants have a dynamic COR. Semi-constrained implants may have a fixed COR, but there may be coupled translation with rotation [41]. A subgroup analysis was performed for those with unrestricted or semi-restricted prostheses to investigate the SALDRS rate between the TDR and ACDF groups in this meta-analysis. The results showed that the SALDRS rate of the TDR group with semi-restricted prosthesis was lower than that of the ACDF group, but without statistical difference, which might be caused by the small number of included studies and patients (only 3 RCTs). In addition, the results also demonstrated that the SALDRS rate of the TDR group with unrestricted prosthesis was significantly lower than that of the ACDF group, and the heterogeneity was low.

This review has some strengths. First, this is the first meta-analysis to evaluate the SALDRS rates after TDR in comparison to ACDF with a minimum follow-up period of 48 months. Second, only RCTs were included in our study. Third, the subgroup analysis by different follow-up periods and different prosthesis designs guarantees consistency and accuracy.

However, the current study also has some limitations influencing its validity. Firstly, only 9 articles were included in this meta-analysis. Since most RCTs comparing TDR and ACDF were designed as non-inferiority studies, the number of RCTs focusing on SALDRS was limited. In the future, more relevant RCTs are needed. Secondly, although the included articles were considered to have a low risk of bias, most studies showed a high risk of bias in incomplete outcome data, and a follow-up rate of over 80% in both groups was observed in only two studies. A review of the literature demonstrated that RCTs investigating the SALDRS rates were insufficient. Therefore, more RCTs focusing on SALDRS with longer-term follow-up are warranted.

## Conclusions

Our study showed that the SALDRS rates of the TDR group were significantly lower than those of the ACDF group at 48–120 months’ follow-up and at different follow-up periods, and the SALDRS rate of the TDR group with unrestricted prosthesis was significantly lower than that of the ACDF group. However, the SALDRS rate of the TDR group with semi-restricted prosthesis was lower than that of the ACDF group, without statistical difference, which may be attributed to the small number of included studies and patients. Hence, more high-quality RCTs with longer-term follow-up are required to achieve a better comparative analysis of the SALDRS rate after TDR and ACDF.

## Data Availability

Datasets are available from the corresponding author on reasonable request.

## Abbreviations

ACDF: Anterior cervical discectomy and fusion
ASD: Adjacent segment disease
CI: Confidence interval
COR: Center of rotation
RCT: Randomized controlled trial
RR: Risk ratio
SALDRS: Symptomatic adjacent-level disease requiring surgery
TDR: Total disc replacement
